# The Alterations in and the Role of the Th17/Treg Balance in Metabolic Diseases

**DOI:** 10.3389/fimmu.2021.678355

**Published:** 2021-07-12

**Authors:** Siwen Zhang, Xiaokun Gang, Shuo Yang, Mengzhao Cui, Lin Sun, Zhuo Li, Guixia Wang

**Affiliations:** Department of Endocrinology & Metabolism, The First Hospital of Jilin University, Changchun, China

**Keywords:** Th17, Treg, chronic inflammation, metabolic disease, obesity, T2DM, MAFLD

## Abstract

Chronic inflammation plays an important role in the development of metabolic diseases. These include obesity, type 2 diabetes mellitus, and metabolic dysfunction-associated fatty liver disease. The proinflammatory environment maintained by the innate immunity, including macrophages and related cytokines, can be influenced by adaptive immunity. The function of T helper 17 (Th17) and regulatory T (Treg) cells in this process has attracted attention. The Th17/Treg balance is regulated by inflammatory cytokines and various metabolic factors, including those associated with cellular energy metabolism. The possible underlying mechanisms include metabolism-related signaling pathways and epigenetic regulation. Several studies conducted on human and animal models have shown marked differences in and the important roles of Th17/Treg in chronic inflammation associated with obesity and metabolic diseases. Moreover, Th17/Treg seems to be a bridge linking the gut microbiota to host metabolic disorders. In this review, we have provided an overview of the alterations in and the functions of the Th17/Treg balance in metabolic diseases and its role in regulating immune response-related glucose and lipid metabolism.

## Introduction

The prevalence of metabolic diseases, such as obesity, type 2 diabetes mellitus (T2DM), and metabolic dysfunction-associated fatty liver disease (MAFLD) continues to increase rapidly in both developed and developing countries. Glucose and lipid metabolism disorders have been associated with chronic inflammation and immune dysregulation, and such aspects form the hallmarks and play important roles in metabolic syndrome. The proinflammatory effects of the innate immunity, represented by macrophages and neutrophils, have been well established. Adaptive immunity, especially mechanisms involving CD4+ T cells, is also critical for the regulation of chronic inflammation and further participates in abnormal energy metabolism. The intricate balance established between proinflammatory T helper 17 (Th17) cells and anti-inflammatory regulatory T (Treg) cells is vital for maintaining immune homeostasis, and has recently attracted increased attention in the regulation of metabolic disorders.

## Th17 and Treg Cells, and the Th17/Treg Balance

According to the specific cytokine profiles and functions documented in previous studies, it is widely recognized that naïve CD4+ T cells may differentiate into one of the lineages of T helper cells (Th1, Th2, and Th17) and Treg cells (1). Interleukin (IL)-17-releasing cells, classified as Th17 cells exhibiting a high expression of the characteristic transcriptional regulator retinoid acid-related orphan receptor γt (RORγt), were first recognized in 2005 (2). The archetypal cytokine IL-17 plays an essential role in neutrophil and macrophage recruitment and mediates inflammatory responses towards infectious agents (3). Additionally, Th17 cells may secrete IL-17A, IL-17F, IL-21, and IL-22, stimulating the secretion of proinflammatory molecules further participating in immunity against bacterial or fungal infections and in the pathogenesis of autoimmune or metabolic diseases (4).

Treg cells are a specific lineage of CD4+ T cells that exert functions to maintain immune homeostasis and to restrict excessive immune responses. Treg cells differentiated peripherally (pTregs) and those derived from the thymus (tTregs) form the two subpopulations of Treg cells *in vivo* (5). Treg cell markers include CD4, CD25, and forkhead box protein P3 (Foxp3). Foxp3 is the master transcription factor of Treg cells and maintains their specific characteristics and functions. Treg cells inhibit naïve T cell activation and prevent the excessive functioning of effector T cells by producing the anti-inflammatory cytokines IL-10 and transforming growth factor (TGF)-β1 (6).

**Figure 1:**
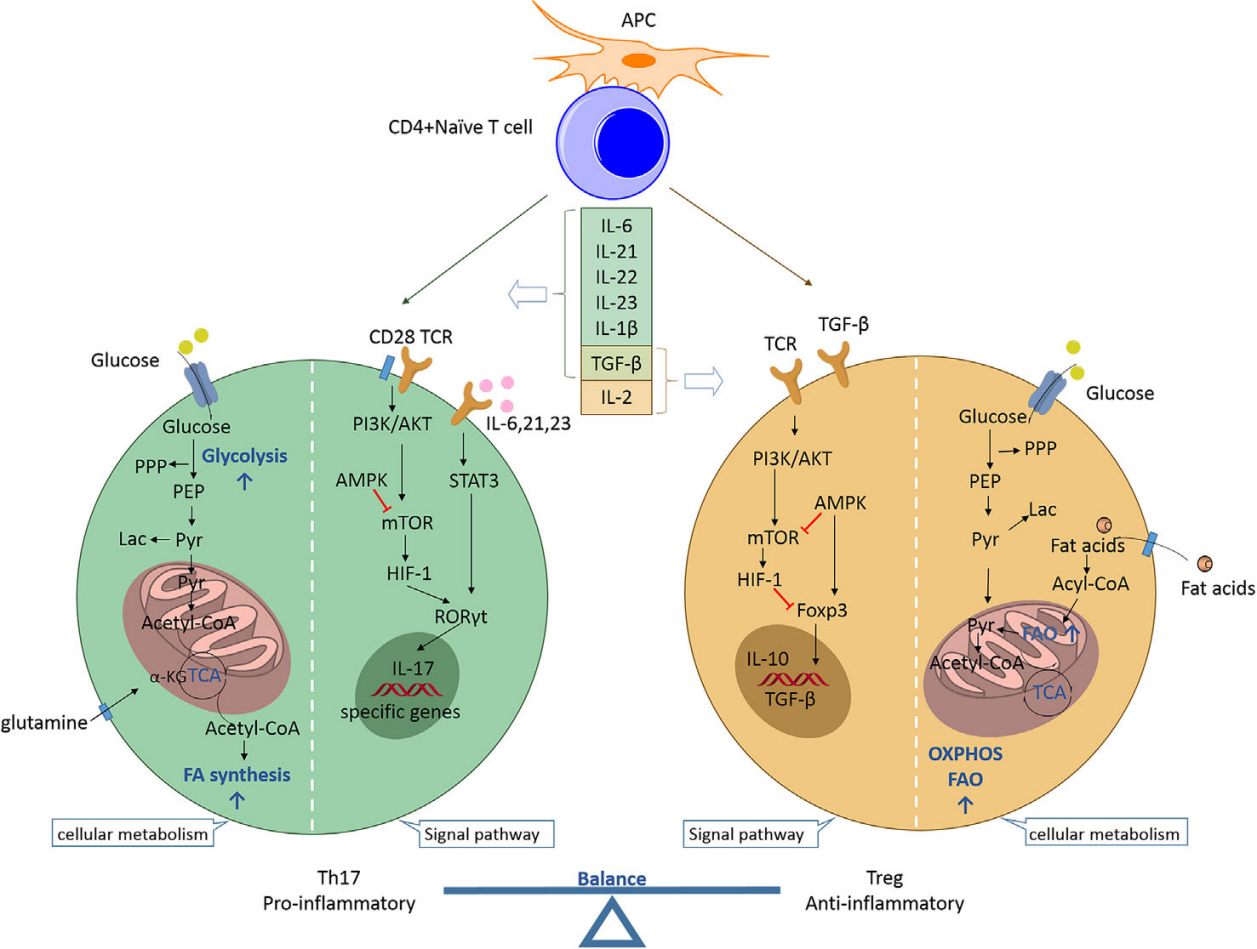
Th17/Treg balance regulated by the cytokine microenvironment and cellular metabolic signaling pathways. Naïve CD4+ T cells may differentiate into T helper cells (proinflammatory Th17 or anti-inflammatory Treg cells) according to specific cytokine profiles. Th17 cell differentiation can be induced by IL-6, IL-21, IL-23, IL-1β, and TGF-β. The proinflammatory cytokines IL-6, IL-21, and IL-23 activate STAT3 to induce *RORC* gene expression and stimulate T cells towards Th17 cell differentiation. RORγt promotes the expression of specific genes (e.g., *CCR6*, *CD161*, *IL17a*, *IL17f*, and *IL23r*) to maintain the phenotype and function of Th17 cells. mTOR is activated by PI3K/Akt signaling after T cell receptor activation and co-stimulation along with naïve T cells. mTOR stimulates HIF-1 to support glycolysis and is indispensable for driving the Th17 phenotype. Additionally, the fatty acid biosynthetic pathway has been shown to regulate Th17 cell differentiation and function. The reverse is true for Treg cell differentiation. IL-2 and TGF-β promote Treg cell differentiation during the development of Treg cells, for which Foxp3 is indispensable. AMPK inhibits mTOR activity and promotes Foxp3-induced OXPHOS and FAO, which allow Treg cells to produce ATP and to generate energy. TGF-β is a developmental factor shared by Th17 and Treg cells and its function is dependent on the coexisting cytokines. Th17, T helper 17; Treg, regulatory T; APC, antigen-presenting cell; IL, interleukin; TGF, transforming growth factor; TCR, T cell receptor; PPP, pentose phosphate pathway; PEP, phosphoenolpyruvate; Lac, lactate; Pyr, pyruvate; α-KG, alpha ketoglutarate; TCA, tricarboxylic acid; FA, fatty acid; PI3K, phosphatidylinositol 3-kinase; Akt, protein kinase B; STAT3, signal transducer and activator of transcription 3; RORγt, retinoid acid-related orphan receptor γt; mTOR, mammalian target of rapamycin; HIF-1, hypoxia inducible factor 1; Foxp3, forkhead box protein P3; AMPK, AMP-activated protein kinase; OXPHOS, oxidative phosphorylation; FAO, fatty acid oxidation.

Th17 and Treg cells represent two distinct phenotypes of CD4+ T cells with completely different functions. Th17 cells are proinflammatory, while Treg cells are anti-inflammatory. The balance established between these two subpopulations is crucial for preventing excessive immune activation, autoimmune responses, and metabolic syndrome pathogenesis. Th17 and Treg cell differentiation arising from naive precursors are mutually linked and can be controlled by the cytokine microenvironment and various metabolic states (**Figure 1**).

### Regulation of Th17/Treg Cell Differentiation and Function by the Cytokine Microenvironment

Th17 cell differentiation can be induced by the cytokines IL-6, IL-21, IL-23, IL-1β, and TGF-β. IL-6 signaling is the first step in the differentiation of Th17 cells derived from naïve CD4+ T cells. Subsequently, IL-23 and IL-21 promote the differentiation and proliferation of Th17 cells (7). Proinflammatory cytokines, such as IL-6, IL-21, and IL-23, activate signal transducer and activator of transcription 3 (STAT3) to stimulate T cells toward Th17 differentiation (8). STAT3 signaling further induces the expression of RORγt, a transcription factor that promotes and maintains the expression of Th17-specific genes (e.g., *CCR6*, *CD161*, *IL17a*, *IL17f*, and *IL23r*) (9). The Th1 cell-specific cytokine interferon gamma (IFN-γ) promotes Th17 differentiation, while the Th2 cell-specific cytokine IL-4 inhibits the differentiation (1). The signaling networks of Th1, Th2, and Th17 mediated by specific cytokines are regulated by Treg cells to maintain immune homeostasis. Foxp3 is indispensable for Treg cell development. Foxp3 inhibits Th17 cell differentiation by establishing direct interactions with RORγt and by inhibiting its binding to DNA, thereby leading to T-cell differentiation into the Treg cell lineage *via* the Th17 transcriptional program (10). Furthermore, TGF-β is a developmental factor shared by Th17 and Treg cells. It induces the expression of both Foxp3 and RORγt, and it drives the differentiation of iTreg cells into Th17 cells in a manner dependent on the presence of proinflammatory cytokines, such as IL-6, IL-1β, and tumor necrosis factor (TNF)-α (11). Thus, the balance established between Treg and Th17 cells is controlled by the action of proinflammatory or anti-inflammatory cytokines. IL-6 plays an important role in determining the direction of the differentiation pathway. Its absence drives the naïve CD4 T cells towards differentiation into the Treg cells, while its presence promotes differentiation into Th17 cells (7).

### Metabolic Control of Th17 and Treg Cells

Glycolysis, glutaminolysis, and fatty acid metabolism are the three main metabolic pathways in CD4+ T cells that function to provide energy. Activated T cells undergo remarkable metabolic changes that are characterized by metabolic reprogramming with increased glycolysis to support cell biosynthesis and function (12). Metabolic reprogramming is necessary during T-cell activation. Th17 cells, as effector T cells of the short-lived inflammatory T cell population, are hypothesized to rely more on glycolysis than the other metabolic pathways. Recent studies have revealed that aerobic glycolysis is indispensable for driving Th17 cell differentiation and function (13). Glycolysis is a series of cytosolic enzymatic reactions that catalyzes the conversion of glucose into pyruvate, thereby generating energy. Aerobic glycolysis is a metabolic process that involves the utilization of glucose to generate lactate with sufficient oxygen (14). It has been reported that glucose metabolism-related genes are highly expressed and expression levels of the intermediates, including pyruvate, lactate, and the pentose phosphate pathway, are enhanced in Th17 cells (15). Furthermore, the action of pyruvate kinase M2, the final rate-limiting enzyme in glycolysis, is necessary for Th17 differentiation (16). Inhibition of glycolysis, such as that with 2-deoxy-d-glucose treatment, may inhibit Th17 cell development and cytokine production (17). Additionally, other metabolic pathways are involved in Th17 cell differentiation. The mechanism underlying glycolysis leading to Th17 cell polarization may be mediated by fatty acid synthesis (FAS). Indeed, Th17 cell function is dependent on fatty acid metabolism, thereby implicating the synthesis of several essential fatty acid derivatives in the regulation of Th17 cell function (18). The key enzyme of *de novo* FAS is acetyl-CoA carboxylase (ACC), which catalyzes the carboxylation of acetyl-CoA into malonyl-CoA. Metabolic profiles of the fatty acid biosynthetic pathway have been shown to enhance Th17 cell differentiation and function (19). Th17 cell polarization is boosted by increased *ACC1* gene expression and RORγt binding to the *IL-17* gene locus. Interestingly, ACC1 modulates RORγt binding to target genes during Th17 cell differentiation but does not affect RORγt expression levels (20). Pharmacological inhibition or T cell-specific deletion of ACC1 has been shown to not only result in blockade of *de novo* FAS, but also cause interference with the metabolic flux of glucose-derived carbon *via* glycolysis and the tricarboxylic acid cycle, thereby attenuating Th17 cell-mediated autoimmune disease in mouse models (21). Additionally, Th17, but not Treg, cell induction *in vitro* depends on glutaminolysis and the upregulation of glutaminase 1 (GLS1) expression to support the tricarboxylic acid cycle and for the catalysis of redox and epigenetic reactions (22). Glutaminolysis is a type of amino acid metabolism that begins with the uptake of extracellular glutamine *via* transporters. This is followed by the conversion of intracellular glutamine to alpha-ketoglutarate (α-KG), which is catalyzed by GLS1, glutamate dehydrogenase, or transaminases (23). GLS1 overexpression and glutamine deprivation studies have confirmed the relationship between glutaminolysis and RORγt expression and its effects exerted on Th17 cells. Chemical or siRNA-mediated inhibition of GLS1 expression has been shown to reduce Th17 differentiation by enhancing mammalian target of rapamycin (mTOR) signaling (22, 24). The inhibition of peroxisome proliferator-activated receptor gamma (PPARγ) agonists on Th17 cells exerts a significant inhibitory effect on glutaminolysis, but not on glycolysis, suggesting that glutaminolysis regulates Th17 cell differentiation independent of glycolysis (25). These findings indicate that there exists a close relationship between Th17 cells and the metabolic pathways.

The energy requirement and metabolic processes of Treg cells are markedly different from those of Th17 cells. Treg cells rely more on oxidative phosphorylation (OXPHOS) and fatty acid oxidation (FAO) than any other processes as a source of fuel to produce ATP and to generate energy (26). During OXPHOS, reactive oxygen species (ROS) are generated, and the total ROS concentration in Treg cells has been found to be significantly greater than that in other T cell subsets in both mice and humans (27, 28). FAO is a key process in fatty acid degradation and an important ATP source. It occurs in the mitochondria, and its key enzyme is carnitine acyl transferase I (CPT1A) that performs the synthesis of acyl carnitine. The fatty acid transporters CPT1A and fatty acid binding protein 5 are expressed in higher levels in Treg cells than those expressed in Th17 cells (29). FAO is required to maintain Treg-cell mitochondrial homeostasis (30). However, FAO is dispensable in Treg cell differentiation. Data obtained from genetic models have shown that Treg cell development and function can occur normally in the absence of CPT1A (31). The mevalonate pathway also plays an important role in Treg cell proliferation by catalyzing the synthesis of sterol isoprenoid intermediates, such as cholesterol (32). Activation of the mevalonate pathway *via* upregulation of its associated gene expression is essential for establishing Treg cell functional competency and for maintaining stability by inducing Treg cell proliferation and by suppressing IFN-γ and IL-17A expression (33). Inhibition of the mevalonate pathway in Treg cells results in a severe decrease in their number, suggesting that this pathway is indispensable for Treg cell survival (34). Activation of the mevalonate pathway promotes Foxp3 expression and stimulates Treg cell proliferation and function by increasing the phosphorylation of Smad3 and by enhancing TGF-β signaling (32). Mevalonate metabolism-driven protein geranylgeranylation and farnesylation orchestrate the differentiation and maintenance of effector Treg cells by serving as rheostats for the immunological receptor, mTORC1, and Rac signaling (35). Interestingly, the preferential pathways employed by Treg cells differ *in vivo* and *ex vivo*. Elevated glycolysis in Treg cell proliferation and activation experiments indicated that the glycolytic pathway provided additional energy *in vitro* (28). Thus, Treg cells depend more on OXPHOS and FAO for *in vivo* functioning and exhibit increased glycolysis *ex vivo*.

Cellular metabolic regulation is intricately associated with immune cell functions and differentiation. Therefore, substances that alter immune cell metabolism may affect Th17/Treg cell differentiation, proliferation, and function. Several chemical compounds or therapeutic strategies improve glucose metabolism and metabolic disorders by changing the percentage of Th17 or Treg cells (**Table 1**). Cytokines and metabolic profiles affect the balance between Th17 and Treg cells *via* different signaling pathways and epigenetic modifications. We would further discuss these pathways and epigenetic modifications extensively to demonstrate the complexity of the relationships existing between them and Th17/Treg balance.

**Table 1:** Chemical compounds or treatment strategies regulate metabolic disorders *via* changing Th17 or Treg percentage.

Reference	Chemical compounds or treatment strategies	Effect	Th17	Treg	Th17/Treg
Cheng et al., 2012 (36)	non-toxic regulatory oligodeoxynucleotides	Reduced obesity-associated insulin resistance	**↓**		
Byun et al., 2013 (37)	Epigallocatechin-3-gallate (EGCG)	Reduced the body weight and fat infiltration in liver tissue while improving serum lipid profiles in diet-induced obesity mice.			**↓**
Liu et al., 2014 (38)	3, 3′-diindolylmethane	Alleviated intra-hepatic inflammation of NASH			**↓**
Kim et al., 2015 (39)	metformin	Improved glucose metabolism and metabolic disorder in mice with high-fat diet-induced obesity.			**↓**
Chang et al., 2015 (40)	SR1555 [1-(4-((49-(1,1,1,3,3,3-hexafluoro-2-hydroxypropan-2-yl)-[1,19-biphenyl]-4-yl)methyl)piperazin-1-yl) ethanone]	Resulted in a modest reduction in food intake accompanied with significant reduction in fat mass, body weight and improved insulin sensitivity of obese diabetic mice.	**↓**		
Aso et al., 2015 (41)	sitagliptin, a DPP-4 inhibitor	Improve glycemic control of T2DM patients	**↓**	**↓**	
Gomes et al., 2016 (42)	hepatic unconventional prefoldin RPB5 interactor	Induce white adipose tissue mediating insulin resistance (IR) and cause NASH.	↑ in liver		
Liu et al., 2017 (43)	OX40-KO	OX40-KO mice exhibited significantly less weight gain and lower fasting glucose levels than those of WT mice		**↑**	
Bao et al., 2017 (44)	PsTag600-FGF21	Dose-dependently reduced body weight, blood glucose, and insulin and lipid levels and reversed hepatic steatosis of NASH	**↓**		
Hong et al., 2017 (45)	Adoptive transfer of *in vitro *differentiated gut-tropic Th17 cells to obese mice	Improve glucose intolerance and insulin resistance	**↑**		
He et al., 2017 (46)	polyene phosphatidylcholine capsules	Attenuating liver inflammatory responses in mice with NAFLD			**↓**
Gilleron et al., 2018 (47)	Rab4b (a small GTPase governing endocytic trafficking) depletion	Specific depletion of Rab4b in T cells causes adipocyte hypertrophy and insulin resistance in chow-fed mice and worsens insulin resistance in obese mice	**↑**	**↓**	
Liu et al., 2018 (48)	Chronic intermittent hypoxia	Accelerates the formation of NASH and fibrosis in mice by high-fat diet administration			**↑**
Gong et al., 2019 (49)	Cajanonic acid A (CAA)	Reduce insulin resistance in HepG2 cells			**↑**
Ding et al., 2019 (50)	glycyrrhizin	Ameliorate lipid metabolism abnormalities of Apoe^–/–^ mice			**↓**
Van Herck et al., 2020 (51)	Adoptive cell transfer of Treg cells.	Exacerbated hepatic steatosis of HFD-fed mice		**↑** in SAT	
Sun et al., 2018 (52)	OX40^-/-^	Decreased liver fat accumulation, lobular inflammation, and focal necrosis after feeding with diets that induce NASH.	**↓**		

### Function of Metabolic Signaling Pathways in Regulating the Th17/Treg Balance

#### The mTOR Signaling Pathway

As a type of phosphatidylinositol 3-kinase-related kinase, mTOR can sense the cell environment and is activated to induce a metabolic shift to support T helper cell generation and function. Both proinflammatory cytokines and metabolic processes, including glycolysis and glutaminolysis, enhance Th17 cell proliferation and differentiation *via* the activation of mTOR signaling (53, 54). Indeed, the mTOR-dependent nutrient-sensing pathway is essential for the differentiation of CD4+ effector T cells (55). Naïve T cells that lack the mTOR complex or those subjected to treatment with the mTOR inhibitor rapamycin do not develop into Th17 cells; however, such cells may exhibit an increase in Foxp3 expression and Treg cell phenotype generation (56). One possible explanation is that mTORC1 inhibits the expression of growth factor independent 1 transcriptional repressor and increases RORγt expression on Th17 cells, thus enhancing their differentiation (57). In contrast, mTOR pathways perform negative regulation of Treg cell differentiation by antagonizing Smad3 and Smad4 during TGF-β signaling or by inactivating Foxo1 and Foxo3 transcription factors (58). Additionally, mTOR deficiency may intensify the sensitivity of naïve T cells to TGF-β, which overcomes the function of STAT3, even in a proinflammatory environment, thereby weakening Th17 cell differentiation (56). However, evidence shows that mTORC1 signaling is a pivotal positive determinant of Treg cell function. Zeng et al. reported that mTORC1 signaling in Treg cells promoted cholesterol/lipid metabolism, with the mevalonate pathway being particularly important for coordinating Treg cell proliferation and for upregulating the expression of suppressive molecules, cytotoxic T lymphocyte antigen 4, and inducible co-stimulators to establish Treg cell functional competency (59). Thus, mTOR signaling affects the Th17/Treg balance, and this warrants further exploration.

The transcription factor hypoxia-inducible factor 1 (HIF1) is another mechanistic molecule of the mTOR signaling that triggers Th17 cell differentiation associated with glycolysis. HIF1α, a known inducer of glycolytic enzymes, is a common component of pathways involved in the regulation of cellular metabolism and is necessary for the expression of glycolytic genes (17). HIF1α plays a role in promoting Th17 cell differentiation and in inhibiting Treg cell development *in vitro* because HIF1α can promote glucose transport and glycolysis at the transcriptional and translational levels (60). HIF1α is a key metabolic programmer that drives Th17 cell differentiation by activating RORγt gene (*RORC*) expression. This phenomenon has been confirmed by ChIP analysis *in vitro* (61). Under conditions of skewed Th17 cell differentiation, HIF1α-deficient CD4+ T cells generate lower proportions of IL-17+ cells and higher proportions of Foxp3+ cells than those generated by HIF1α+ CD4+ T cells *in vitro* (17). As for Treg cell development, HIF1α inhibits cell differentiation by binding to Foxp3 and by triggering its degradation (61). These findings suggest that HIF1α contributes to an establishment of the balance between Th17 and Treg cells.

#### The Phosphatidylinositol 3-Kinase (PI3K)/Protein Kinase B (Akt) Pathway

The PI3K/Akt pathway is located upstream of mTOR, and is crucial to several aspects of cell growth and survival. mTOR is activated by PI3K/Akt signaling after T cell receptor activation and co-stimulation with naïve T cells. This pathway is closely related to the generation of effector T and Treg cells by regulating metabolic processes. The activation of the PI3K signaling pathway increases glucose transporter expression and facilitates its transfer to the cell membrane, and enhances glycolysis, thereby skewing the Th17/Treg balance (62). It has been reported that inhibition of PI3K/Akt or genetic knockout of the delta isoform of PI3K can suppress Th17 cell and promote Treg cell differentiation (63). Furthermore, appropriate PI3K functioning is essential for the maintenance of Treg cell generation and function, whereas excessive PI3K activity seems to be detrimental. The effect and mechanism of action of PI3K on the Th17/Treg balance may be multifaceted, including promotion of mTOR activity and glucose uptake, mediated by distinct PI3K isoenzymes (64). Indeed, the effects of PI3K/Akt signaling exerted on the function and frequency of Treg cell populations and their importance in Th17 cell differentiation remain unknown. Further investigation of specific PI3K inhibitors may help provide data for the development of potential immune-modifying drugs for the regulation of the Th17/Treg balance.

#### The AMP-Activated Protein Kinase (AMPK) Pathway

AMPK is a heterotrimeric kinase complex consisting of a catalytic α subunit, a regulatory β subunit, and an AMP-binding γ subunit. The γ subunit of AMPK acts as a sensor of cellular AMP/ATP levels and is activated under low-energy conditions. AMPK is associated with glucose uptake and glycolysis in multiple cell types (65). AMPK activation plays an antagonistic role in mTOR-dependent signaling pathways. Defective functioning of AMPK results in an increase in mTOR activity accompanied by the upregulation of glycolysis and an elevated production of effector cytokines (66). In contrast, AMPK is known to be particularly active in Treg cells (67). Activation of AMPK seems to drive the differentiation of naïve T cells into iTreg cells over Th17 cells by enhancing FAO (65). The direct activators of AMPK help provide the necessary material and result in the localization of FAO using various mechanisms, such as fatty acid uptake and generation in the mitochondria, to enable robust enhancement of Treg cell expansion (68). Similarly, the indirect AMPK activator metformin suppresses T cell proliferation and inhibits the differentiation of Th17 cells while promoting the development of Treg cells in a dose-dependent manner *in vitro* (69).

### Epigenetic Regulation

The effect of epigenetic modification on the Th17/Treg balance is associated with the expression of RORγt and Foxp3 and is regulated by the metabolic signaling pathways. Several cytokines have been reported to induce changes in H3K4me3 levels at the *Il17a* and *Il17f* promoters in murine Th17 cells, with TGF-β driving an increased H3K4me3 presence at both the promoters and with IL-23 driving a reduced H3K4me3 presence at the *Il17a* promoter (70). p300, known as histone acetyltransferase, is reportedly important for HIF1-mediated gene activation. HIF1α can bind with RORγt at the IL-17A promoter for the recruitment of p300 to generate a loose chromatin structure and to activate Th17 locus transcription (71). Histones H3 and H4 located around the IL-17A promoter region have been shown exhibit increased levels of acetylation in HIF1+ T cells than those observed in HIF1-deficient T cells under Th17-promoting conditions (61). The Treg cell transcriptional program can be inhibited *via* degradation of Foxp3 protein with HIF-1/RORγt/p300 activation (10). Foxp3 expression and the induction of a specific DNA hypomethylation signature during development is necessary for Treg cells. The epigenetic regulator ubiquitin-like with plant homeodomain and RING finger domain 1 is essential for the maintenance of methyl-DNA marks that establish stability of Treg cell identity by repressing effector T cell transcriptional programs (72). α-KG, as an intermediate product of glycolysis and glutaminolysis, can increase the methylation of the Treg cell-specific demethylated region within the *FOXP3* locus, thereby inhibiting Treg cell differentiation (73). The equilibrium established between phenotypic plasticity and stability of Th17 and Treg cells is defined by the fine-tuned transcriptional and epigenetic events necessary to ensure stable expression of characteristic genes such as *RORC* and *FOXP3*.

Therefore, Th17/Treg imbalance can be a consequence of changes occurring in the cytokine microenvironment and metabolic disorders through complex mechanisms. It also plays a crucial role in the development and progression of metabolic diseases. In the following section, we have mainly focused on obesity and MAFLD to elucidate the role of the Th17/Treg balance in metabolic diseases and its relationship with the gut microbiota.

## The Imbalance of the Th17/Treg Cell Ratio in Visceral Chronic Inflammation and Adipogenesis

**Figure 2:**
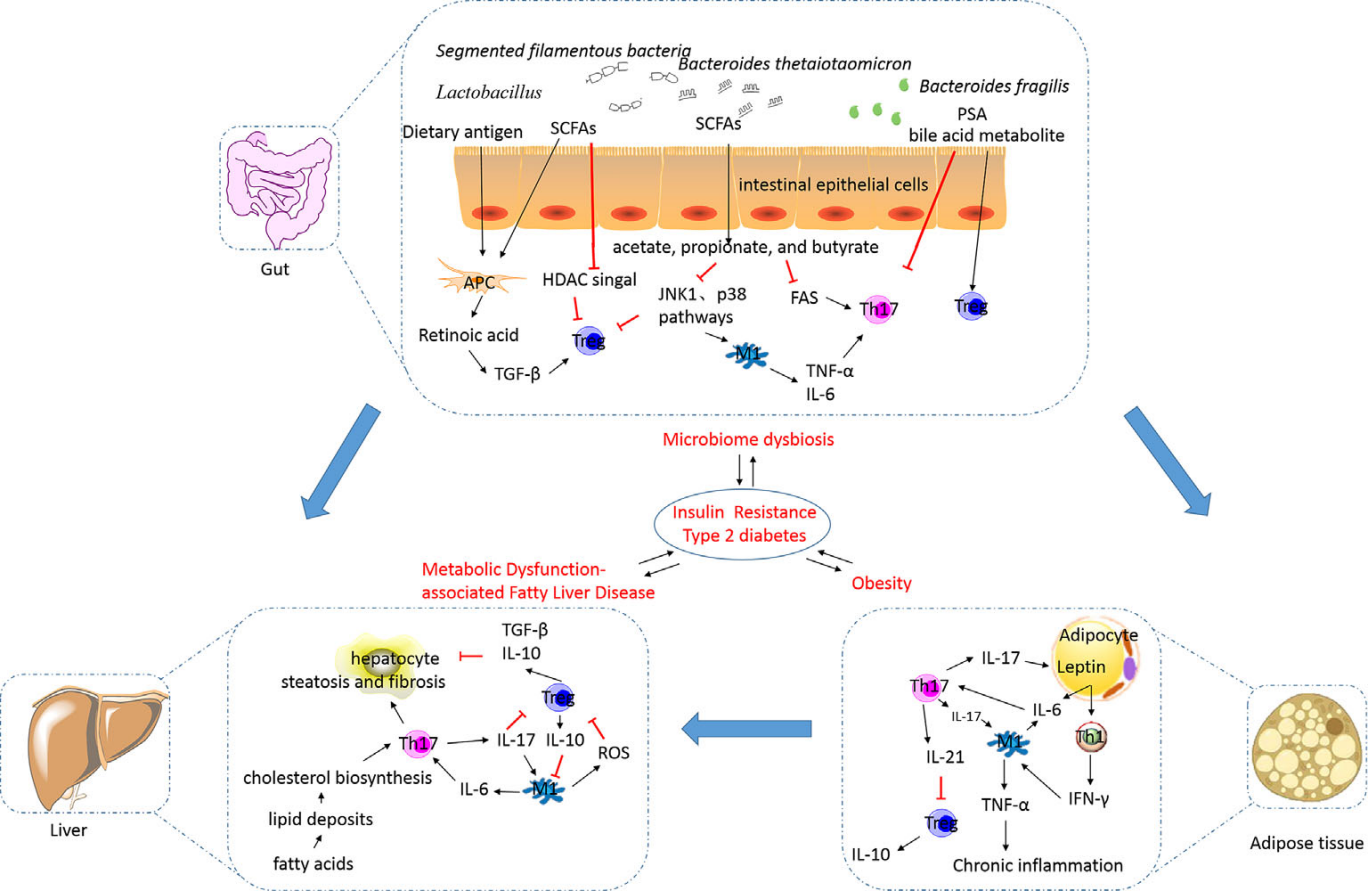
The alterations in and the roles of the Th17/Treg balance in metabolic disorders. Excess nutrition can result in the development of obesity and metabolic dysfunction-associated fatty liver disease, and this phenomenon is reportedly influenced by the gut microbiota. This results in the development of insulin resistance, diabetes, metabolic syndrome, and related cardiovascular complications (their relationships were showed by blue arrows). In obesity, adipocytes can secrete proinflammatory cytokines, including IL-6, to increase the number of Th17 cells. Th17 cells stimulate macrophages towards an inflammatory signaling cascade. IL-17 secreted by Th17 cells targets adipocytes and participates in the proinflammatory signaling. IL-21 secreted by Th17 cells can inhibit Treg cell differentiation and function. The decreased number of Treg cells in visceral adipose tissues is inversely correlated with the body mass index and plasma leptin levels. Increased release of free fatty acids by white adipose tissue causes hepatocyte injury and leads to the synthesis of proinflammatory cytokines. IL-17A increases cytokine production in hepatocytes and macrophages, thereby inducing steatosis and fibrosis. M1 macrophages can inhibit the function of Treg cells *via* ROS-induced apoptosis. TGF-β and IL-10 secreted by Treg cells are involved in the exertion of anti-inflammatory effects. The intestinal microbiota is essential for the development of obesity and plays an important role in regulating NAFLD progression. Translocation of bacteria and bacterial products activates inflammasomes and stimulates proinflammatory cytokines to cause a shift in the Th17/Treg balance. *Lactobacillus reuteri, Bacteroides fragilis*, *Bacteroides thetaiotaomicron*, *Clostridium*, and *Faecalibacterium prausnitzii* promote Treg cell differentiation. *Segmented filamentous bacteria* are necessary for the development of the gut Th17 cells. SCFAs, as metabolites of microbes such as acetate-, propionate-, and butyrate-producing microbes, can limit Th17 cell and promote Treg cell differentiation. Bile acid transformation mediated by the gut bacteria can increase Foxp3 expression in Treg cells. Th17, T helper 17; Treg, regulatory T; IL, interleukin; TGF, transforming growth factor; SCFA, short-chain fatty acid; PSA, polysaccharide A; HDAC, histone deacetylase; JNK, c-Jun N-terminal kinase; FAS, fatty acid synthesis; TNF-α, tumor necrosis factor alpha; IR, insulin resistance; T2DM, type 2 diabetes mellitus; NAFLD, non-alcoholic fatty liver disease; ROS, reactive oxygen species; IFN-γ, interferon gamma.

Obesity, a state occurring due to excess nutrition, is associated with low-grade chronic inflammation and the secretion of pathogenic proinflammatory mediators. This results in the development of diabetes, metabolic syndrome, and related cardiovascular complications (74). It is evident that obesity impairs immune function, while the affirmation that an altered immune system underlies the onset of obesity warrants further investigation. An excess presence of lipids and glucose can directly exert impacts on Th17 and Treg cell activation and differentiation *via* the modulation of nutrient sensor activity (**Figure 2**). In obesity, adipocytes can increase the number of Th17 cells by secreting proinflammatory cytokines, including IL-6 (75). The polarization of the Th17 phenotype has been shown to be critical for sustaining adipose tissue (AT) inflammation and for contributing towards the development of other chronic inflammatory diseases (76). Polarization of macrophages towards M1, neutrophil influx into AT, activation of Th1 and Th17 cells, and increased levels of proinflammatory cytokines are associated with stimulation of the inflammation cascade in AT (77). Additionally, a high-fat diet (HFD) promotes Th17 cell differentiation depending on the ACC1-mediated *de novo* FAS, including increased IL17A, IL23R, LTB4R1, and CCR6 expression (20). Several experiments in human or animal models have shown the occurrence of changes in Th17 cell percentages in patients with obesity or T2DM. Peripheral blood mononuclear cells (PBMCs) derived from patients with obesity and T2DM have shown an increase in the number of Th17 cells up to varying degrees (78–85). Animal data corroborate this finding (42). The degree of increase in the number of Th17 cells in the AT is correlated with the hemoglobin A1c (HbA1c) level and blood glucose regulation. Interestingly, it has also been reported that the Th17 cell percentage remains unchanged in the PBMCs of patients with obesity or T2DM, and in the visceral adipose tissue (VAT) of animals fed with an HFD (41, 82, 86–88). It is postulated that the percentage of Th17 cells changes gradually and the anomalies are distributed across various tissues with the development of obesity, leading to inconsistency of data generated by studies targeted on obese patients with different course of disease.

Th17 cells play a critical role in proinflammatory cytokine production, adipogenesis, and glucose homeostasis during the development of obesity (**Figure 2**). IL-17, a major effector cytokine produced by Th17 cells, has been shown to regulate the mediation between the AT and Th17-mediated immune responses (89). Increased IL-17A expression has been detected in obese individuals and mice and is associated with increased Th17 cell infiltration in AT (90). In obese individuals, IL-17A enhances the production of proinflammatory cytokines, such as IL-6, in VAT. This increase in IL-6 levels may favor Th17 cell differentiation, based on mechanisms discussed previously. Th17 cells seem to obstruct the insulin receptor signaling pathway and to reduce insulin sensitivity by enhancing the secretion of IL-17 and IL-22, which contributes to metabolic dysfunction (91). IL-17A exacerbates insulin resistance and promotes the infiltration of Th17 cells in cooperation with TNF-α in the AT (92). Additionally, IL-17 decreases adipogenesis by downregulating the expression of specific proadipogenic transcription factors, including PPARγ and CCAAT-enhancer-binding protein α (88). Blockade of cyclooxygenase 2 (COX-2) expression can reduce the inhibitory effect of IL-17A on adipogenic differentiation (93), implicating COX-2 expression in the inhibitory effect of IL-17A. However, animal studies have shown that an IL-17A deficiency increases diet-induced obesity in mice and prevents the development of disorders related to glucose metabolism (88). Pestel et al. reported that IL-17A blockade fails to lead to the recovery of adipogenesis and insulin response (94); however, the detailed mechanisms remain poorly defined. The inconsistency in determining the relationship between Th17 cells and IL-17 cytokines implies that other sources of IL-17. Recent studies have shown that γδ T cells are also a predominant source of IL-17 in white AT in obesity (95). In general, the functions of Th17 cells, especially those in the insulin-sensitive tissues, and their pathological relevance are largely elusive and warrant further investigation.

As obesity progresses, the number of Treg cells is decreased, concurrently with increased Th17/Treg balance. Animal research has shown that VAT of lean mice is highly enriched with Treg cells, which are markedly reduced in number under obese or insulin-resistant conditions (38, 46, 96, 97). Several studies conducted on obese and/or T2DM patients also support the above-mentioned phenomenon (79, 87, 98–100). Moreover, the decreased number of Treg cells in VATs is inversely correlated with the body mass index and plasma leptin levels (101). Chronic inflammation and metabolic disorders are the principal reasons for a decrease in the number of Treg cells. IL-21 expression is elevated in AT of obese individuals and reduces the number of Treg cells in VAT by downregulating the expression of the transcriptional regulator IFN regulatory factor 4. As obesity develops, adipocytes express increased levels of leptin, which correlates with expression of the major histocompatibility complex II by adipocytes, strongly inhibiting Treg cell accumulation in VAT (102). VAT-resident Treg cells possess a transcriptionally unique phenotype and antigen-receptor repertoire compared with their counterparts in the spleen and lymph nodes (103). Treg cells in VAT overexpress C-C motif chemokine receptors 1 and 2 and secrete IL-10, and this occurrence is different from that observed in the conventional Treg cell populations (104). This suggests that the accumulation of Treg cells in VAT depends on the establishment of interactions with the local antigen-presenting cells.

The number of VAT-resident Treg cells is negatively correlated with insulin resistance in obesity (96). Obesity is accompanied by the development of hyperinsulinemia. In obesity, the function of Treg cells is impaired by insulin *via* Akt/mTOR activation (105). Treg cells function in the regulation of whole-body metabolism to maintain insulin sensitivity in the visceral tissue by limiting M1 macrophage infiltration and cytokine IL-10 secretion. IL-10 inhibits the function of TNF-α for downregulating glucose transporter 4 expression and for impairing insulin action in adipocytes (106). Furthermore, TGF-β secreted by Treg cells has been reported to decrease body weight and insulin resistance (107). The level of TGF-β has been shown to be lower in obese individuals than that in healthy individuals (108). The effects of Tregs that control inflammation induced by obesity and adipogenesis are dependent on PPARγ and Foxp3 (103). Imbalance of the Th17/Treg cell ratio has been suggested to serve as an important mechanism of obesity. On one hand, the imbalanced differentiation of Th17 cells and Treg cells exists in proinflammatory cytokine microenvironment of obesity. Proinflammatory cytokines, including IL-6 secreted by adipocytes and macrophages, drive the naïve CD4 T cells towards differentiation into the Th17 cells. On the other hand, the immunosuppressive effect of Treg cells on Th17 cells is reduced. Treg cells can inhibit Th17cells by specifically upregulating hydroxy prostaglandin dehydrogenase (HPGD), thereby preventing local inflammation and systemic IR (109). This function was weakened with the decrease of Treg cells in obesity. The increased Th17 cells and decreased Treg accelerate proinflammation. Thus, imbalance of the Th17/Treg cell ratio can mediate the occurrence of obesity-related inflammation and metabolic disorders.

## The Role of Th17/Treg Balance in Metabolic Associated Fatty Liver Disease (MAFLD)

MAFLD, referred to as non-alcoholic fatty liver disease/non-alcoholic steatohepatitis (NAFLD/NASH), is a chronic liver disease that may progress from simple fatty liver to hepatic steatosis, presented by a spectrum of metabolic syndromes. The pathogenesis of NASH is complex and involves the establishment of crosstalk between the gut, AT, and liver. Moreover, NASH pathogenesis is mediated by the action of cytokines, adipokines, and the cellular immune system (110). Enhanced release of free fatty acids by white AT causes hepatocyte injury by increasing triglyceride synthesis and storage in the liver (111). It is assumed that excessive activation of cholesterol biosynthesis and accumulation of lipid deposits in hepatocytes leads to the synthesis of proinflammatory cytokines, ultimately resulting in hepatitis (112). The sustained inflammatory activity caused by Kupffer cells, neutrophils, and specific lymphocyte subsets infiltrating the liver is critical for MAFLD development. Th17 cells and their secreted IL-17 are important components of this process. Liver damage is associated with an increase in IL-23 levels, which induces a Th17 cellular response (110). Furthermore, increased lipid and cholesterol levels in MAFLD regulate the differentiation of T lymphocytes into Th17 cells by the expression of nuclear receptors and genes, such as sterol regulatory element binding protein 1c and ATP-binding cassette transporter A1 (113).

Treatment of MAFLD with hydroxymethylglutaryl coenzyme A reductase inhibitors, which are considered as lipid-lowering agents inhibiting cholesterol synthesis, can also affect the Th17/Treg balance (34). An increased number of Th17 cells has been reported in the liver tissues of patients with NASH compared with healthy individuals (114). Similar outcomes have also been noted in mice fed with an HFD (38, 46, 97, 112, 115–117). These findings revealed that IL-17 signaling activation (Th17 cells and IL-17 production) might be central to the pathogenesis of NAFLD and NASH. Several *in vivo* experiments have shown increases in steatosis upon treatment with IL-17, while blockade of IL-17 or knockdown of *IL17* decreases steatosis and confers protection to animals against NASH development (118). In contrast, another study suggested that IL-17 functional inhibition was responsible for an increase in steatosis in the liver (119). One possible reason is that IL-17 production is not limited to Th17 cells. Other cells capable of producing IL-17 include natural killer T cells, γδ T cells, and group 3 innate lymphoid cells (120). This implies that IL-17 signaling may also play a protective role in NAFLD *via* indirect mechanisms. Notably, IL-17A expression increases lipid uptake and cytokine production in hepatocytes (112). Th17 cells participate in hepatic stellate cell fibrogenic machinery by promoting IL-17-induced collagen production in a c-Jun N-terminal kinase- and STAT3-dependent manner (121). Therefore, the role and mechanism of action of Th17 cells in NAFLD development and pathogenesis warrant further investigation.

Expression of the cytokine IL-17 inhibits Treg cell differentiation through TGF-β signaling pathways and promotes Th17 cell differentiation by stimulating IL-6 production, thereby enhancing Th17/Treg imbalance (122). Thus, Treg cells may play a role in regulating inflammatory processes, in contrast to Th17-mediated responses, in NASH. The number of liver-residing Treg cells is reduced in NAFLD animal models (38), and local ROS-induced apoptosis of Treg cells may be one of the underlying reasons. It has been shown that induction of Treg cells in patients with NASH decreases aspartate transaminase activity and results in the exertion of positive biological effects on the liver (123). Adoptive transfer of Treg cells decreases TNF-α expression and attenuates HFD-induced hepatic inflammation (124). TGF-β and IL-10, secreted by Treg cells, seem to be involved in the exertion of this antifibrotic effect (125, 126). However, contradictory findings have been reported in humans with an increased Treg cell number in liver steatosis (127, 128). Hypercholesterolemia elevates Foxp3 expression and Treg cell proportion in the liver-resident T cell population (129). The increase in the number of Treg cells is also probably due to the protective feedback against prevailing dyslipidemia. The proportion of Treg cells shows positive correlation with low-density lipoprotein cholesterol and apolipoprotein B in MAFLD patients. The presence of cholesterol and lipoprotein may activate inflammation and Treg cell differentiation, but cannot prevent inflammation. The Treg subset of the human liver is usually characterized indirectly. This confirms that the source and function of liver-resident Treg cells is essential for clarifying the difference in Treg cell function in NAFLD between animal models and humans.

## The Role of Th17/Treg Cells in the Regulation of Glucose and Lipid Metabolism by the Gut Microbiota

Imbalances in the gut microbiota have been considered to contribute to the development of metabolic disease states, even before the occurrence of systemic inflammation. Changes in bacterial diversity and specific components have been observed in the earlier stages of metabolic diseases (130). Experimental HFD-induced animal models have shown that the intestinal microbiota is essential for the development of obesity and plays an important role in regulating NAFLD progression (131). Supplementation of probiotic bacteria, *Lactobacillus*, in mice fed with an HFD can significantly reduce fat accumulation. Similar results have been confirmed in clinical trials wherein probiotic *Lactobacillus-*based treatment improved body composition and lowered visceral fat levels in obese patients (132). Accordingly, the gut microbiota is a new pathogenic factor of obesity and T2DM. However, the mechanisms underlying the gut microbiota regulating glucose and fat metabolism remain unclear. The immune system may participate in this mechanism. The gut microbiota establishes interaction with the local immune environment to regulate one another. Recent studies have confirmed that the intestinal microbiome may exert influence on the Th17/Treg balance. Translocation of bacteria and bacterial products can result in the activation of inflammasomes and stimulation of proinflammatory cytokine production through toll-like receptor and nucleotide oligomerization domain-like receptor signaling, thereby activating the adaptive immune response and shifting the Th17/Treg balance (133). In lean and healthy individuals, the anti-inflammatory cytokines TGF-β, IL-25, and IL-33 secreted by the intestinal epithelial cells maintain a favorable environment for Treg cell differentiation (133). Moreover, treatment with the purified probiotic microbe *Lactobacillus reuteri* prevents weight gain *via* IL-10 production by Treg cells (134). The metabolite polysaccharide A derived from *Bacteroides fragilis* has been reported to confer protection against intestinal inflammation mediated by the decreased production of IL-17 by immune cells residing in the intestine and promotion of Treg cell differentiation (135). *B. fragilis* promotes the induction of Foxp3+ Tregs and promote IL-10 secretion, which can rebalance biased Th17/Treg levels and increase the suppressive capacity of Foxp3+ Tregs, thus mitigating pathological inflammation (136). Similar results have been reported for *Bacteroides thetaiotaomicron*, which has been found to regulate the nuclear-cytoplasmic shuttling of PPAR-γ. *Clostridium* and *Faecalibacterium prausnitzii* have also been reported to favor Treg cell development (137). In contrast, certain segmented filamentous bacteria are necessary for the development of the gut Th17 cells (138). Therefore, disruption of the intestinal microbiota contributes to alterations in the Th17/Treg balance, regulating immune activation and the development of chronic inflammation.

The gut microbiota has been reported to participate in carbohydrate metabolism through several metabolic pathways. Short-chain fatty acids (SCFAs), as metabolites of microbes such as acetate-, propionate-, and butyrate-producing microbes, play essential roles in Th17 and Treg cell induction (139). SCFAs can influence the cellular energy status, and the ATP/ADP levels through their integration in tricarboxylic acid as Acetyl-CoA (43–121, 132). Both butyrate and propionate have been reported to inhibit FAS *via* deactivation of ACC1, thereby limiting Th17 cell and promoting Treg cell differentiation (140). Furthermore, The mechanism of SCFAs regulating T cell differentiation involves G-protein-coupled receptor signaling and the mTOR pathway in a cytokine milieu-dependent manner (141). SCFAs can also perform remodeling of the T cell epigenome to favor Treg cell differentiation and further lead to the exertion of anti-inflammatory effects (142). Butyrate affects Treg cell differentiation mediated by histone H3 acetylation at the *FOXP3* locus (142). Furthermore, butyrate, along with acetate and propionate, is reported to promote the generation and function of pTreg cells through inhibition of histone deacetylase (HDAC) and enhancement of histone H3 acetylation at the *FOXP3* promoter (143). It is reported that butyrate produced by *F. prausnitzii* decrease Th17 differentiation by inhibiting HDAC3 and c-Myc-related metabolism in T cells (144). Succinate inhibits expression of the ten-eleven translocation family of DNA methylases in Treg cells, thereby blocking the expression of suppressive genes without altering cell proliferation (145). Bile acid transformation mediated by the gut bacteria directly modulates the Th17/Treg balance. The bile acid metabolite dehydrolithocholic acid inhibits Th17 cell differentiation by directly binding to RORγt, and lithocholic acid enhances Treg cell differentiation through increased Foxp3 expression (146). In general, the role of the Th17/Treg balance in the gut microbiota regulating the immune responses and host metabolism is important. Therapeutic measures associated with the gut microbiota variety may contribute to improving chronic inflammation associated with obesity by regulating the Th17/Treg balance.

## Conclusions and Future Perspectives

Recent human and animal studies have shown the important roles played by CD4+ T cells in chronic inflammation, with a shift from Treg to Th17 cell differentiation that is related to the development of obesity. This highlights the establishment of a Th17/Treg balance between the immune response and insulin resistance, obesity, and MAFLD. Although the roles of Th17 and Treg cells in metabolic disorders have been widely investigated, the results of these clinical trials are inconsistent. The targets of Th17/Treg cells in metabolic diseases are widespread and include the ATs, muscles, liver, and intestine. Indeed, evidence has suggested that there may be a connection between the gut microbiota and the host metabolic state *via* Th17/Treg cell activation and migration. However, the detailed mechanisms are not fully understood, and further studies are warranted. Owing to the ethical limitations with human specimens, it is difficult to study the migration of Th17 and Treg cells dynamically. Therefore, animal models, including murine models, have been commonly studied. However, certain differences exist between humans and mice in terms of metabolic and immune physiological functions. Whether the data obtained from mice may represent a real phenomenon in humans requires extensive validation. Accordingly, immune system-based humanized mice may be used to deduce a selective approach to resolve this issue.

Medication or treatment strategies targeting Th17 and Treg cells to improve metabolic diseases are considered as research hotspots. Few research findings are summarized in **Table 1**. Thus, targeting of Th17 and/or Treg cells using small molecules or inhibitors affecting intracellular glucose and lipid metabolism may be considered an advanced approach to treating obesity-induced disorders. Based on the existing knowledge, conduction of further research focusing on the mechanisms underlying the roles of Th17 and Treg cells in metabolic disease pathogenesis may provide novel therapeutic avenues for metabolic diseases.

## Author Contributions

Conceptualization: ZL and GW. Methodology and literature arrangement: SZ and XG. Writing - original draft preparation: SZ and MC. Writing - review and editing: SZ, SY and LS. Funding acquisition: ZL and GW. Supervision: ZL and GW. All authors contributed to the article and approved the submitted version.

## Funding

The present study was supported by grants from Engineering Technology Research Center for Personalized Precision Diagnosis and Treatment, Science and Technology Department of Jilin Province (20170623005TC was awarded to GW); Innovation Capability Project of Jilin Provincial Development and Reform Commission (grant no.2017C019 was awarded to GW); Department of Science and Technology of Jilin Province (grant no. 20190701040GH was awarded to ZL).

## Conflict of Interest

The authors declare that the research was conducted in the absence of any commercial or financial relationships that could be construed as a potential conflict of interest.
